# Feel the Noise: Relating Individual Differences in Auditory Imagery to the Structure and Function of Sensorimotor Systems

**DOI:** 10.1093/cercor/bhv134

**Published:** 2015-06-19

**Authors:** César F. Lima, Nadine Lavan, Samuel Evans, Zarinah Agnew, Andrea R. Halpern, Pradheep Shanmugalingam, Sophie Meekings, Dana Boebinger, Markus Ostarek, Carolyn McGettigan, Jane E. Warren, Sophie K. Scott

**Affiliations:** 1 Institute of Cognitive Neuroscience; 2 Faculty of Brain Sciences, University College London, London, UK; 3 Center for Psychology, University of Porto, Porto, Portugal; 4 Department of Psychology, Royal Holloway University of London, London, UK; 5 Department of Otolaryngology, University of California, San Francisco, USA; 6 Department of Psychology, Bucknell University, Lewisburg, USA

**Keywords:** auditory imagery, auditory processing, fMRI, supplementary motor area, voxel-based morphometry

## Abstract

Humans can generate mental auditory images of voices or songs, sometimes perceiving them almost as vividly as perceptual experiences. The functional networks supporting auditory imagery have been described, but less is known about the systems associated with interindividual differences in auditory imagery. Combining voxel-based morphometry and fMRI, we examined the structural basis of interindividual differences in how auditory images are subjectively perceived, and explored associations between auditory imagery, sensory-based processing, and visual imagery. Vividness of auditory imagery correlated with gray matter volume in the supplementary motor area (SMA), parietal cortex, medial superior frontal gyrus, and middle frontal gyrus. An analysis of functional responses to different types of human vocalizations revealed that the SMA and parietal sites that predict imagery are also modulated by sound type. Using representational similarity analysis, we found that higher representational specificity of heard sounds in SMA predicts vividness of imagery, indicating a mechanistic link between sensory- and imagery-based processing in sensorimotor cortex. Vividness of imagery in the visual domain also correlated with SMA structure, and with auditory imagery scores. Altogether, these findings provide evidence for a signature of imagery in brain structure, and highlight a common role of perceptual–motor interactions for processing heard and internally generated auditory information.

## Introduction

Imagine the voice of a close friend when you laugh together, or a piano playing your favorite song. Auditory imagery is a complex process by which an individual generates and processes mental images in the absence of sound perception—“hearing with the mind's ear.” Auditory mental images can be so vivid that they resemble the real experience of hearing, and they can be as accurate as representations arising directly from sensory input (Janata 2012). They facilitate several cognitive and motor processes. In music performance, for instance, imagery supports action planning, formation of expectations about upcoming events, and interpersonal coordination (Keller 2012; Novembre et al. 2014). Functional neuroimaging studies have shown that the network of brain regions engaged during auditory imagery minimally includes the superior temporal gyri (STG), parietal, motor, and premotor cortices, the inferior frontal gyrus, and the supplementary motor area (SMA) (Shergill et al. 2001; Herholz et al. 2012; Zvyagintsev et al. 2013; for a meta-analysis, McNorgan 2012).

The involvement of STG in auditory imagery has been suggested to reflect the reconstruction of sound-like representations via higher order cortical mechanisms, contributing to the subjective experience of “hearing” (Kraemer et al. 2005; Zatorre and Halpern 2005). The superior parietal cortex is associated with the manipulation of imagined auditory events, for example when the task requires participants to mentally reverse the notes of a melody (Zatorre et al. 2010). Frontal regions are assumed to underlie general control, working memory, retrieval, and semantic processes (Zvyagintsev et al. 2013). The SMA and premotor cortices seem to be directly involved in generating auditory images (Halpern and Zatorre 1999; Herholz et al. 2012), implicating an intimate link between sensorimotor and imagery processes. Consistent with the idea that auditory–motor interactions may be involved in auditory imagery, in a functional magnetic resonance imaging (fMRI) study, Kleber et al. (2007) showed that the premotor cortex and SMA are active both when professional singers overtly sing an Italian aria and when they are asked to imagine the act of singing as vividly as possible without performing any movements. Functional imaging work has additionally revealed that auditory imagery recruits brain networks that also respond to heard auditory information (Zatorre et al. 1996; Kosslyn et al. 2001; Zatorre and Halpern 2005; Herholz et al. 2012). For instance, Zatorre et al. (1996) asked participants to make pitch judgments about words taken from familiar tunes in an imagery condition, in which there was no auditory input, and in a perceptual condition, in which participants could actually hear the song. Common activations were found across conditions despite the differences in input, including the temporal and frontal lobes, the supramarginal gyrus, midbrain, and SMA.

We have a good picture of the functional networks that are active during auditory imagery tasks, but a common aspect to many of the available studies is that findings are based on group averages—similarities across individuals are privileged over interindividual differences so that general processes may be inferred. Less is known about the predictors of individual differences in how people experience auditory images, or about which neural systems account for these differences. These questions matter, as behavioral data reveal considerable variability in how well individuals perform on tasks that engage imagery abilities, e.g., judging whether or not a final probe note of a scale is mistuned when the initial notes were played but the remaining ones had to be imagined (Janata 2012). People also vary widely in how vividly they experience auditory mental images, as measured by self-report on the Bucknell Auditory Imagery Scale (BAIS; Pfordresher and Halpern 2013). In that study, higher vividness of imagery predicted better accuracy in a pitch imitation task in which participants reproduced sequences of pitches, suggesting that the sensorimotor components of imagery play a role in planning and guiding vocal imitation. In 2 fMRI studies, individual differences in the BAIS correlated with blood oxygen level-dependent (BOLD) responses in the right superior parietal cortex during a task involving mental reversal of melodies (Zatorre et al. 2010), and in the right STG, right dorsolateral prefrontal cortex and left frontal pole during imagery of familiar tunes (Herholz et al. 2012).

Crucial to the understanding of interindividual differences in imagery is the question of whether they are determined by the local structure of gray matter. A growing number of studies indicates that individual differences in a range of basic and higher order cognitive functions are reflected in brain structure, as measured using techniques such as voxel-based morphometry (VBM) and diffusion tensor imaging (for a review, Kanai and Rees 2011). Differences in brain structure have been reported among groups of experts, such as musicians (Gaser and Schlaug 2003), taxi drivers (Woollett and Maguire 2011), and phoneticians (Golestani et al. 2011), as well as in samples from the general population. For instance, among people with no particular expertise, increased gray matter volume in the left thalamus predicts enhanced ability to adjust to degraded speech (Erb et al. 2012) and, in the right anterior prefrontal cortex, it predicts the ability to introspect about self-performance during perceptual decisions (Fleming et al. 2010).

In the present study, we examine for the first time whether differences in brain structure predict differences in how auditory images are subjectively experienced. Gray matter volume was measured using VBM, and auditory imagery was evaluated in terms of perceived vividness, as well as in terms of perceived control over mental representations, that is, the ease with which people can change or manipulate representations (Pfordresher and Halpern 2013; Halpern 2015). Two additional novel questions were addressed. First, we combined VBM and fMRI approaches to investigate whether the structural predictors of imagery co-localize with systems that also play a role in the processing of heard auditory information. Importantly, in addition to looking at co-localization, we examined possible co-variation between interindividual differences in auditory imagery and in the patterns of online functional responses to auditory input. Electrophysiological studies have shown similar modulations of the N100 component by imagery and sensory-based auditory processes (Navarro-Cebrian and Janata 2010a), and imaging studies have reported common activations during imagery and auditory processing (Zatorre et al. 1996; Kosslyn et al. 2001; Zatorre and Halpern 2005; Herholz et al. 2012), a result suggestive of converging mechanisms. However, because co-localization does not necessitate shared function (e.g., Woo et al. 2014), more direct evidence for links between the processing of heard and internally generated auditory information is needed. Second, in an additional VBM study, we aimed to determine the extent to which the structural predictors of auditory imagery reflect the operation of mechanisms that are specialized to auditory information. To that end, links with visual imagery were investigated. Research on imagery is typically confined to a single modality, but some fMRI studies suggest that whereas the STG may play an auditory-specific role, the SMA, premotor, parietal, and prefrontal regions may be involved in imagery within and beyond the auditory domain, forming a modality-independent “core” imagery network (Daselaar et al. 2010; McNorgan 2012; Burianová et al. 2013). Therefore, the current study takes advantage of combining behavioral with structural and functional measures to shed new light on the neural underpinnings of interindividual differences in auditory imagery, and on how these differences may reflect mechanisms shared with sensory-based processing and the operation of supramodal processes.

## Materials and Methods

### Participants

Seventy-four participants were included in the study looking at the structural correlates of auditory imagery (*M*_age_ = 42.61, SD = 17.11; range = 20–81; 40 female). None reported a diagnosis of neurological or psychiatric disorders. Written informed consent was collected and ethical approval was obtained from the UCL Research Ethics Committee. All structural scans were reviewed by a neurologist to identify anatomical abnormalities that could affect their suitability for VBM; this led to the exclusion of 2 participants of the 76 initially included. No participants had significant cognitive impairment (all participants aged ≥50 years completed the Montreal Cognitive Assessment, *M*_score_ = 28, max 30; SD *=* 1.68; range = 25–30; www.mocatest.org). The participants' age range was wide because these data were collected as part of a larger project on neurocognitive ageing. All participants completed the forward condition of the digit span test of the Wechsler Adult Intelligence Scale (WAIS-III, Wechsler 1997; average number of digits correctly recalled = 7.08; SD *=* 1.21; range = 4–9). Short-term memory is highly correlated with working memory and intelligence (Colom et al. 2008) and, therefore, it was used as a proxy for general cognitive abilities. Thirty participants had some degree of musical training (*M*_years of training_ = 6.03, SD = 4.47; range = 1–20).

From the 74 participants, 56 completed the fMRI study examining brain responses during auditory processing (*M*_age_ = 47.05, SD = 17.23; range = 20–81; 31 female).

Forty-six participants took part in the follow-up VBM study looking at the links between auditory and visual imagery (44 of them also participated in the first VBM study; *M*_age_ = 47.13, SD = 17.83; range = 20–81; 24 female).

### Materials

#### Individual Differences in Imagery

To assess auditory imagery, we used the BAIS (Pfordresher and Halpern 2013; Halpern 2015), a self-report measure that includes 2 14-item subscales. The first subscale focuses on “vividness” of imagery: participants are asked to generate a mental image of the sound described in each item, and to rate its subjective clarity in a 7-point scale (1 = no image present at all; 7 = as vivid as actual sound), for example, “consider ordering something over the phone; the voice of an elderly clerk assisting you”; “consider attending classes; the slow-paced voice of your English teacher.” The second subscale focuses on “control” of imagery: participants are asked to generate mental images corresponding to pairs of items, and to consider how easily they can change the first image to the second image (1 = no image present at all; 7 = extremely easy to change the item), for example, “consider ordering something over the phone; image a—the voice of an elderly clerk assisting you; image b—the elderly clerk leaves and the voice of a younger clerk is now on the line.” Most of the items cover vocal and musical sounds, with only a minority of them focusing exclusively on environmental sounds (3 items in each subscale; e.g., the sound of gentle rain). The BAIS has appropriate psychometric properties, including high internal reliability, a coherent factor structure, and no association with social desirability (Halpern 2015). It has been used in behavioral (Pfordresher and Halpern 2013; Gelding et al. 2015) and fMRI studies (Zatorre et al. 2010; Herholz et al. 2012).

To assess visual imagery, we used the Vividness of Visual Imagery Questionnaire (VVIQ; Marks 1973). In this task, participants are given 4 hypothetical scenarios and generate 4 mental images corresponding to different aspects of each scenario, forming 16 items in total (e.g., contour of faces; color and shape of trees; attitudes of body of a friend or relative). Responses are provided on a scale from 1 (perfectly clear and vivid as normal vision) to 5 (no image at all), that is, lower scores correspond to higher vividness, unlike the BAIS in which the direction of the scale is reversed. For ease of interpretation, scores were inverted so that higher scores correspond to higher vividness both in the auditory (BAIS) and visual domains (VVIQ). The VVIQ is the most frequently used self-report measure of vividness of visual imagery. It has appropriate internal reliability (Kozhevnikov et al. 2005; Campos and Pérez-Fabello 2009) and correlates with brain responses during visual perception and imagery (Cui et al. 2007).

#### Auditory Stimuli

The auditory stimuli used in the fMRI study consisted of 5 types of human vocal sounds. These included vowels spoken with a neutral intonation (e.g., prolonged “a”), laughter, screams, and sounds of pleasure and disgust (retching sounds). Similar to imagery processes, these vocal communicative signals are known to engage auditory systems, as well as sensorimotor and control systems involved in higher order mechanisms and social behavior (Warren et al. 2006; McGettigan et al. 2015). The 5 sound types were matched for duration (*M*_duration_ = 1018 ms; SD *=* 326), and 20 different examples of each were included in the experiment (they were generated by 8 different speakers, 4 women; for further details about the stimuli, Sauter et al. 2010; Lima et al. 2013). A sixth condition, intended as an unintelligible distractor set, consisted of sounds created by spectral rotation of a selection of the original vocal sounds. Rotated sounds were generated by inverting the frequency spectrum around 2 kHz, using a digital version of the simple modulation technique described by Blesser (1972). The acoustic signal was first equalized with a filter (essentially high-pass) that gave the rotated signal approximately the same long-term spectrum as the original. This equalizing filter (33-point finite impulse response [FIR]) was constructed based on measurements of the long-term average spectrum of speech (Byrne et al. 1994), although the roll-off below 120 Hz was ignored, and a flat spectrum below 420 Hz was assumed (Scott, Rosen, et al. 2009; Green et al. 2013). The equalized signal was then amplitude modulated by a sinusoid at 4 kHz, followed by low-pass filtering at 3.8 kHz. Spectral rotation retains the acoustic complexity of human sounds while rendering them unintelligible. Rotated sounds are used in numerous imaging studies of vocalizations and speech perception (Scott et al. 2000; Narain et al. 2003; Warren et al. 2006; Okada et al. 2010; Evans et al. 2014).

### MRI Acquisition and data Processing

MRI data were acquired using a 32-channel birdcage headcoil on a Siemens 1.5-T Sonata MRI scanner (Siemens Medical, Erlangen, Germany). High-resolution anatomical images were acquired using a *T*_1_-weighted magnetization prepared rapid acquisition gradient echo sequence (repetition time = 2730 ms, echo time = 3.57 ms, flip angle = 7°, slice thickness = 1 mm, 160 sagittal slices, acquisition matrix = 256 × 224 × 160 mm, voxel size = 1 mm^3^). Echo-planar fMRI images were acquired with repetition time = 9 s, TA = 3 s, echo time = 50 ms, flip angle = 90°, 35 axial slices, 3 mm^3^ in-plane resolution, using a sparse-sampling routine in which sounds were presented in the silent gap between brain acquisitions (Hall et al. 1999).

#### Voxel-Based Morphometry

The structural images were subjected to VBM, as implemented in SPM8 (Wellcome Trust Centre for Neuroimaging, UK). SPM8 provides an integrated routine that combines segmentation into different tissue classes, bias correction, and spatial normalization in the same model (New Segment). After being re-oriented into a standard space (via manual alignment along the anterior–posterior commissure), each participant's *T*_1_-weighted image was segmented into gray matter, white matter, and cerebrospinal fluid. Diffeomorphic Anatomical Registration was performed through exponentiated lie algebra (DARTEL) for nonlinear intersubject registration of the gray and white matter images (Ashburner 2007). This involves iteratively matching the images to a template generated from their own mean, that is, sample-specific gray and white matter templates were generated.

Because we were interested in differences across subjects in the absolute “amount” (volume) of gray matter, the spatial normalization step was implemented with modulation in order to preserve the total amount of gray matter signal in the normalized partitions. This is necessary as the process of normalizing images introduces volumetric changes in brain regions; for the structural images to be aligned and matched across subjects, expansions, or contractions may be needed due to individual differences in brain structure. To account for the amount of expansion and contraction, the modulation step adjusts the normalized gray matter values by multiplying by its relative volume before and after spatial normalization (e.g., if a participant's temporal lobe doubles in volume during normalization, the correction will halve the intensity of the signal in this region; Mechelli et al. 2005). The resulting values at each voxel thus denote the absolute amount of tissue that is gray matter at that location, after having adjusted for the confounding effects of nonlinear warping. While an analysis based on modulated data (implemented in the current study) tests for variability in the amount of gray matter, an analysis without modulation tests for variability in “concentration” of gray matter (Ashburner and Friston 2000; Mechelli et al. 2005). Finally, the images were transformed to Montreal Neurological Institute (MNI) stereotactic space (voxel size = 1.5 mm^3^), and smoothed using a 10 mm full-width half-maximum (FWHM) isotropic Gaussian kernel. VBM provides a mixed measure of cortical surface (or cortical folding) as well as cortical thickness, unlike surface-based approaches, that emphasize measures of thickness derived from geometric models of the cortical surface (e.g., Hutton et al. 2009). Further work is needed to specify the exact cellular basis of local differences in the amount of gray matter as measured by VBM. However, these are assumed to potentially reflect variability in the number and size of neurons or glia, or in axonal architecture (May and Gaser 2006; Kanai and Rees 2011).

Multiple regressions were conducted on the smoothed gray matter images. At the whole-brain level, per-participant auditory imagery scores were entered into a general linear model, including age, gender, total gray matter volume (Peelle et al. 2012), short-term memory, and years of musical training as nuisance variables in the design matrix to regress out any potential confounding effects related to them. Musical training was included because this has been shown to correlate with vividness of auditory imagery (Pfordresher and Halpern 2013), with the acuity of mental auditory images in performance-based tasks (Janata and Paroo 2006; Navarro-Cebrian and Janata 2010a,2010b), as well as with differences in brain structure (Gaser and Schlaug 2003). Regressing out variability in short-term memory is important to ensure that correlations between imagery and gray matter cannot be attributed to nonspecific factors linked to general cognitive functioning. While a memory component may be involved in imagery (e.g., Navarro-Cebrian and Janata 2010b), the need to control for the general cognitive demands of the tasks has been highlighted (Halpern et al. 2004; Zatorre and Halpern 2005), and this is of special relevance in the context of an off-line self-report measure as the one used here. Any voxels showing gray matter intensity <0.05 were excluded using an absolute masking threshold to avoid possible edge effects around the border between gray matter and white matter. Statistical maps were thresholded at *P* < 0.005 peak-level uncorrected, cluster corrected with a family-wise error (FWE) correction at *P* < 0.05, while accounting for nonstationary correction (Ridgway et al. 2008). In addition to whole-brain analysis, more sensitive region of interest (ROI) analyses were conducted within regions for which we had a priori hypotheses, based on a recent activation likelihood estimation meta-analysis of fMRI studies of imagery across modalities (McNorgan 2012). We covered 2 networks identified by this meta-analysis, one derived from auditory imagery studies only (8 studies), and the other one from studies involving imagery across multiple modalities (65 studies). When a region was reported in both networks, we choose the coordinate of the auditory-specific one. Table 1 presents the list of ROIs and corresponding MNI coordinates. Statistical significance within these ROIs was assessed using small volume correction (Worsley et al. 1996) at a threshold of *P* < 0.05 (FWE corrected), within spheres with 12 mm radius centered at each of the coordinates.

**Table 1:** VBM results for vividness of auditory imagery on regions previously identified to be functionally associated with auditory imagery and general imagery

Region of interest	VBM results								
Area	MNI coordinates			Peak coordinates			*Z* score	*t*_(1,67)_	*P*
	*x*	*y*	*z*	*x*	*y*	*z*			
**Auditory imagery network**									
R superior temporal gyrus	64	−30	9						n.s.
L inferior frontal gyrus	−48	24	−5						n.s.
	−51	17	9						n.s.
L putamen	−21	−1	4						n.s.
L superior temporal gyrus	−60	−38	15						n.s.
L precentral gyrus	−52	1	47						n.s.
L supramarginal gyrus	−58	−38	28						n.s.
R inferior frontal gyrus	56	38	2						n.s.
L supplementary motor area	−1	−14	53	−4	−24	52	3.22	3.36	0.03
	−8	1	69	9	−9	73	3.26	3.40	0.03
**General imagery network**									
L inferior parietal lobule	−30	−56	52	−28	−55	43	3.2	3.34	0.03
	−38	−38	46						
L superior parietal lobule	−16	−62	54						n.s.
R superior parietal lobule	20	−66	54	21	−61	51	3.27	3.41	0.02
R medial superior frontal Gyrus	6	20	44	14	17	48	3.47	3.65	0.01
L middle frontal gyrus	−30	0	56	−35	−7	63	2.98	3.10	0.05

Note: The column “MNI coordinates” shows the coordinates of ROIs, taken from a meta-analysis of imagery studies (McNorgan 2012); anatomical labels for each ROI were determined based on these coordinates, using the SPM Anatomy Toolbox v1.8. Small volume correction was used within 12-mm spheres centered at each of the coordinates. *P* values are FWE corrected (*P* < 0.05) and the obtained peak locations within each sphere are presented (column “peak coordinates”). R, right; L, left; n.s., no local maxima exceeded the specified threshold.

#### fMRI Procedure and Analyses

Functional and structural data were acquired on the same day. Participants were told that they would hear different kinds of sounds, and that they should listen attentively to them. They listened passively to the sounds and were asked to perform a vigilance task consisting of pressing a button every time a “beep” was presented. The sounds were presented in 2 runs of 140 echo-planar whole-brain volumes; each run lasted 21 min. The first 3 volumes from each run were discarded to allow longitudinal magnetization to reach equilibrium. Auditory onsets occurred 5.5 s (±0.5 s jitter) before the beginning of the following whole-brain volume acquisition. On each trial, participants listened to 2 randomly selected sounds of the same type. The sounds were presented in a pseudo-randomized order for each participant, and we ensured that no more than 3 trials of the same type were consecutively presented. All 120 sounds were presented twice per run (plus 9 vigilance and 8 rest/silence trials per run). Sounds were presented using Psychtoolbox (Brainard 1997) via a Sony STR-DH510 digital AV control center (Sony, Basingstoke, UK) and MRI-compatible insert earphones (Sensimetrics Corporation, Malden, MA, USA).

Data were analyzed using SPM8. Functional images were realigned to the first image, unwarped, co-registered to the structural image, and spatially normalized to MNI space using the parameters acquired from segmentation (Ashburner and Friston 2005); they were resampled to 2-mm^3^ voxels and smoothed with a 10-mm FWHM Gaussian kernel. The hemodynamic response was modeled using a first-order FIR filter with a window length equal to the time taken to acquire a single volume. At the first level, the 5 types of vocal sounds, the unintelligible rotated sounds, and the vigilance trials (and 6 movement regressors of no interest) were entered into a general linear model. The rest/silence trials were used as an implicit baseline. At the second level, a one-way repeated-measures analysis of variance (ANOVA) was conducted using contrast images from the first level to identify brain regions in which the magnitude of responses varied as a function of the type of human vocalization; separate contrast images for each of the 5 types of intelligible sounds versus rest baseline were entered in this model (for a similar approach, Warren et al. 2006). The results are presented at an uncorrected threshold of *P* < 0.005 peak level, with nonstationary correction of *P* < 0.05 at cluster level for the whole-brain analysis.

To examine whether the neural systems involved in imagery co-localize with those involved in auditory processing, ROI analyses were conducted focusing on the regions shown to predict auditory imagery in the VBM study (at whole-brain and ROI levels); small volume correction was used at a threshold of *P*_FWE_ < 0.05, within spheres with 12 mm radius, centered at the peak of the clusters. Among these ROIs, when the one-way repeated-measures ANOVA revealed an effect, a more sensitive multivariate Representational Similarity Analysis was also conducted (Kriegeskorte et al. 2008). This analysis was conducted to directly explore whether there is an association between interindividual differences in the specificity of neural representations of heard vocal sounds and variation in self-report auditory imagery ratings. This was achieved by extracting data from the whole-brain t-statistic maps of each of the 5 types of intelligible vocal sounds relative to the resting baseline, and Pearson product-moment correlating these maps with each other. We used t-maps because, as they combine the effect size weighted by error variance for a modeled response, they provide higher classification accuracy in multivariate analyses; results are not unduly influenced by large, but highly variable response estimates (Misaki et al. 2010). In each participant, the correlation coefficients reflecting the relationship between neural responses to each of the 5 conditions with every other condition were converted to a *z* value using a Fisher transformation so as to conform to statistical assumptions (normality) required for parametric statistical tests. These values were averaged to provide a summary statistic for each participant, a higher value reflecting higher similarity between neural responses, that is, lower discrimination between conditions; and a lower value reflecting lower similarity between neural responses, that is, higher discrimination between conditions or more distinct representations. These values were then Pearson product-moment correlated with ratings of auditory imagery.

## Results

### Neuroanatomical Predictors of Individual Differences in Auditory Imagery

There were large individual differences in auditory imagery ratings: For the total imagery scale, ratings ranged between 2.5 and 7 (*M =* 5.12; SD = 0.87); on the Vividness subscale, they ranged between 2.86 and 7 (*M* = 4.96; SD = 0.95); and on the Control subscale, they ranged between 2 and 7 (*M =* 5.28; SD = 0.95). Consistent with previous evidence (Pfordresher and Halpern 2013), vividness and control of imagery were highly correlated with each other (*r* = 0.68, *P* < 0.001). No significant associations were found between imagery and age (total imagery scale, *r* = −0.18, *P* = 0.13; vividness subscale, *r* = −0.14, *P* = 0.25; control subscale, *r* = −0.19, *P* = 0.11), suggesting that these processes are relatively stable across the adult life span. Shapiro–Wilk tests confirmed that the ratings were normally distributed (*P*’s > 0.13).

**Figure 1.**
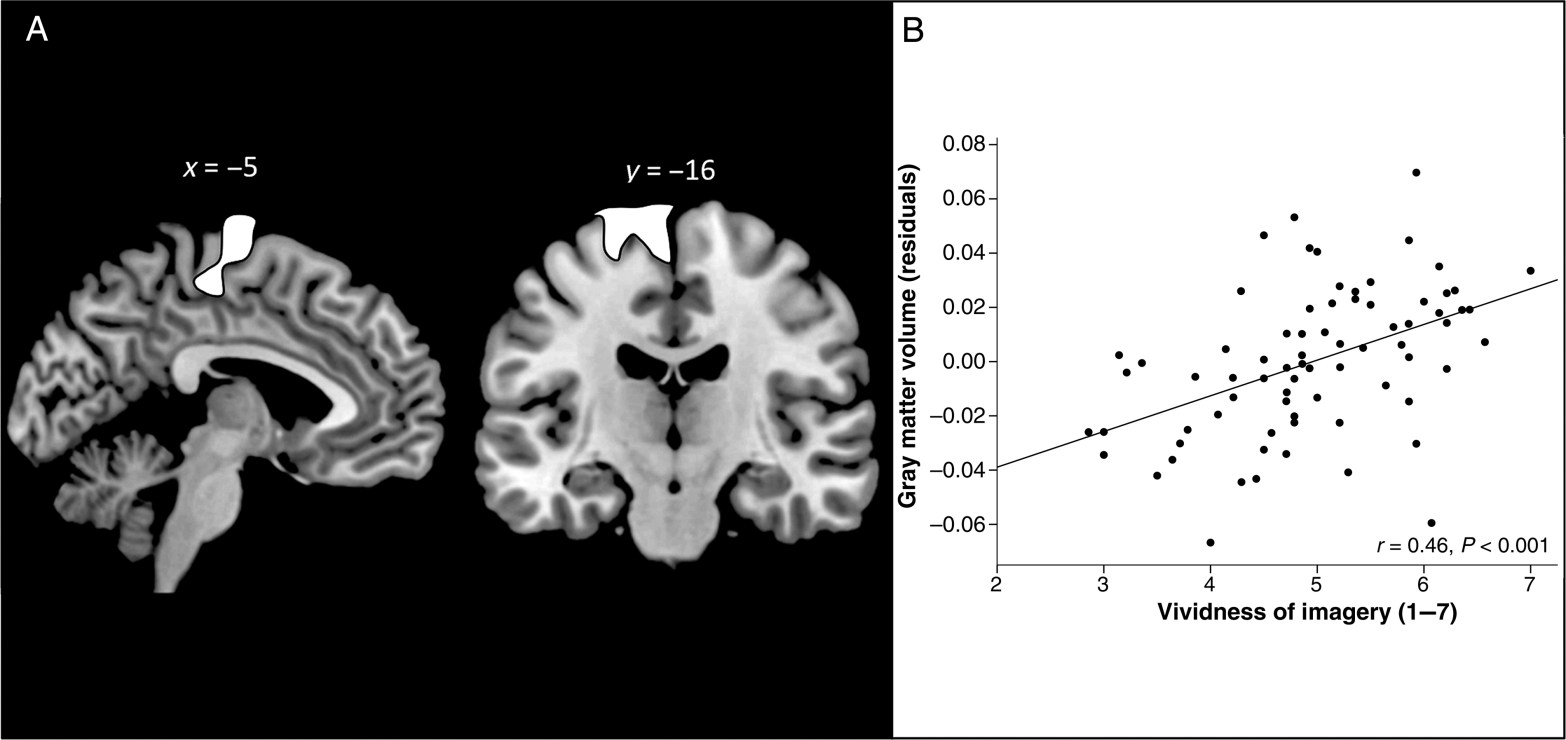
Association between gray matter volume and vividness of auditory imagery. (*A*) Cluster with peak in left SMA showing a significant positive correlation with vividness of auditory imagery in whole-brain analysis. Statistical maps were thresholded at *P* < 0.005 peak-level uncorrected, cluster corrected with a FWE correction (*P* < 0.05). (*B*) Scatterplot showing the association between vividness ratings and adjusted gray matter volume within the cluster depicted in (*A*).

The goal of this experiment was to determine whether individual differences in how people perceive auditory images can be predicted from differences in brain morphology. A first whole-brain analysis focusing on the total imagery ratings (average of the 2 scales) revealed that higher ratings correlated with larger gray matter volume in a cluster with a peak voxel in the left paracentral lobule, extending to the right paracentral lobule, left precuneus, and left superior frontal gyrus (cluster size = 3369 voxels, *P*_FWE_ = 0.03; MNI coordinate for peak voxel: *x* = −8, *y* = −12, *z* = 69, *t*_(1,67)_ = 3.63, *Z* = 3.45, *P* < 0.001 uncorrected). No associations were found between higher imagery ratings and decreased gray matter (for the negative contrast, lowest *P*_FWE_ = 0.43). To directly investigate the structural predictors of each of the 2 auditory imagery components, whole-brain analyses were also conducted on vividness and control ratings separately (we refrained from including the 2 subscales in the same design matrix because they were very highly correlated with each other). For individual differences in control of imagery, no clusters survived correction, either for positive or for negative correlations (lowest *P*_FWE_ = 0.26). For vividness of imagery, on the other hand, a positive correlation was found with regional gray matter volume in a cluster with a peak voxel situated within the left SMA, extending to the left and right paracentral lobules (cluster size = 3531 voxels, *P*_FWE_ = 0.03; MNI coordinate for peak voxel: *x* = −6, *y* = −13, *z* = 67, *t*_(1,67)_ = 3.57, *Z* = 3.40, *P* < 0.001). This cluster is shown in Figure 1, along with a scatterplot between gray matter residuals and vividness scores (*r* = 0.46, *P* < 0.001). No results were found for negative correlations (lowest *P*_FWE_ = 0.84). We extracted gray matter residuals within this SMA cluster and observed that the correlation with higher vividness of imagery remained significant after regressing out variability accounted for by the other subscale, control of imagery (partial correlation, *r* = 0.34, *P* = 0.003). This indicates that the role of this structure for vividness of imagery cannot be reduced to nonspecific factors (e.g., confidence of participants in their judgments or temporal processing), as these would be similarly engaged across subscales.

ROI analyses, using small volume correction, were also conducted within regions hypothesized to be involved in auditory and domain general imagery generation, as identified by a recent meta-analysis of fMRI studies of imagery (McNorgan 2012). We found positive correlations between gray matter volume and vividness of auditory imagery within 5 ROIs. Two of them are part of the auditory imagery network, and they partly overlap with the SMA cluster revealed by the more conservative whole-brain analysis (see Table 1 for full anatomical and statistical details). The other three are part of the general imagery network: one in left inferior parietal lobule, one in right medial superior frontal gyrus, and one in left middle frontal gyrus. Additionally, a negative correlation was found between vividness of auditory imagery and the amount of gray matter in the right superior parietal lobule. Similar analyses focusing on control of imagery ratings revealed a marginally significant association between higher control and increased gray matter volume within the left SMA ROI (MNI coordinate for peak voxel within ROI: *x* = −11, *y* = −9, *z* = 72, *t*_(1,67)_ = 3.11, *Z* = 3, *P*_FWE_ = 0.05), and a negative association in the right medial superior frontal gyrus ROI (MNI coordinate for peak voxel within ROI: *x* = 6, *y* = 15, *z* = 33, *t*_(1,67)_ = 3.12, *Z* = 3.01, *P*_FWE_ = 0.05).

### Functional Responses to Heard Auditory Information

**Figure 2.**
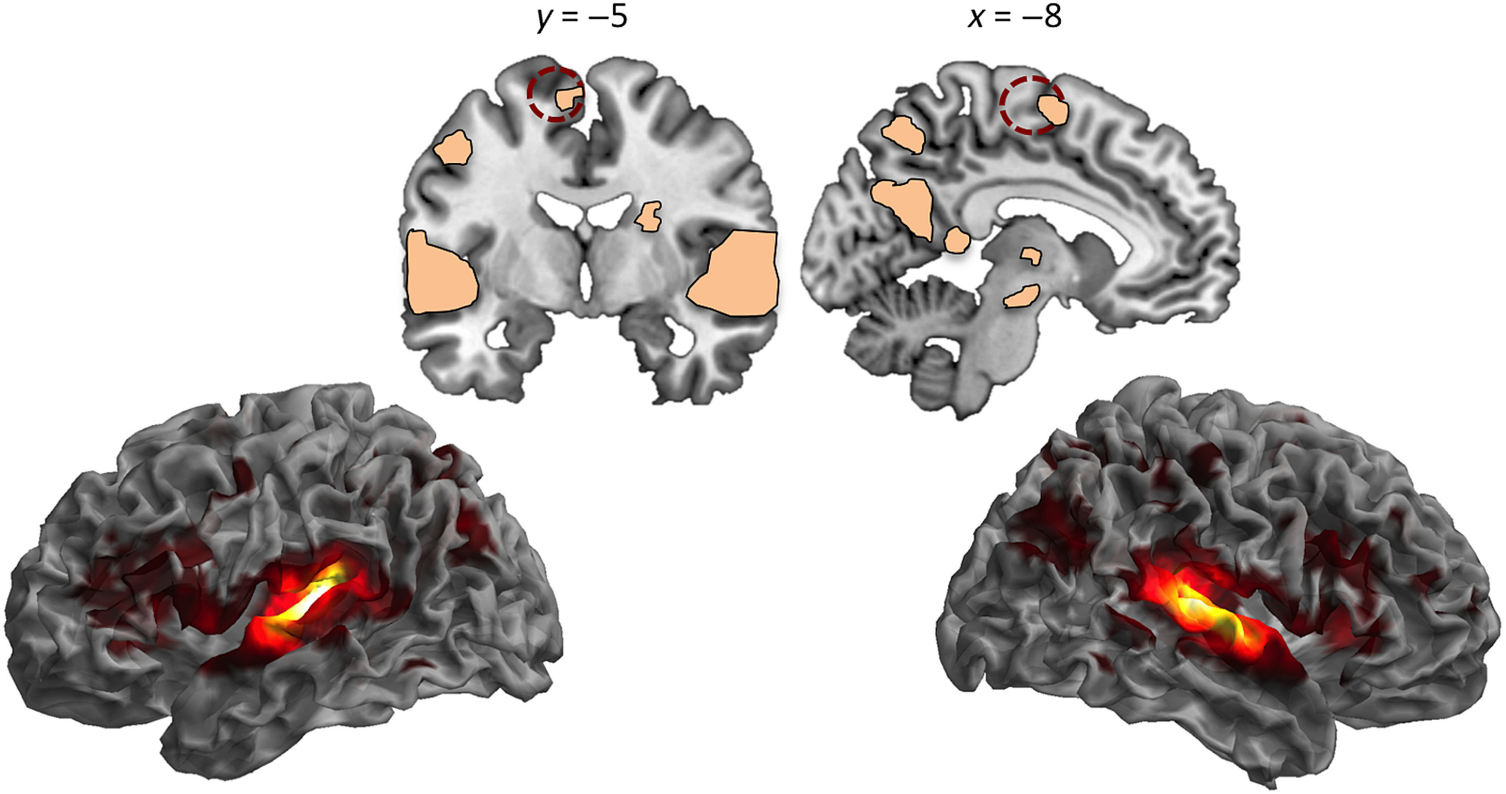
Brain regions in which BOLD responses were modulated by sound type during the processing of heard auditory information. The dotted dark red circle denotes a 12-mm sphere centered at the peak of the SMA cluster where the amount of gray matter was shown to correlate with auditory imagery (VBM study); this sphere was used for the representational similarity analysis looking at the links between representational specificity of heard sounds and vividness of imagery. For visualization purposes, activation maps were thresholded at *P* < 0.005 peak-level uncorrected (full details of activated sites are presented in Table 2).

In the whole-brain analysis, significant modulations of neural responses as a function of sound type were found in a number of brain regions, shown in Figure 2 and listed in Table 2. Consistent with earlier work using similar stimuli (e.g., Warren et al. 2006; McGettigan et al. 2015), activations were largely bilateral and included the STG, precentral and prefrontal cortices, parietal regions, cuneus and precuneus, insula, and thalamus.

**Table 2:** Brain regions showing significant modulations of BOLD responses as a function vocalization type during auditory processing

Region	fMRI results						
	# Voxels	MNI coordinates			*Z* score	*F*_4,220_	*P*
		*x*	*y*	*z*			
R superior temporal gyrus	10 842	60	−24	8	>8	72.85	<0.001
R superior temporal gyrus		62	−14	2	>8	63.28	
R primary auditory cortex		40	−26	12	>8	55.64	
R insula lobe		34	24	4	5.96	13.16	
R inferior frontal gyrus		44	16	28	5.72	12.25	
R inferior parietal cortex		46	−36	48	3.77	6.31	
R inferior parietal cortex		64	−32	42	3.67	6.05	
R postcentral gyrus		38	−36	50	3.65	6.01	
R inferior temporal gyrus		52	−50	−8	3.49	5.64	
R supramarginal gyrus		68	−30	34	3.48	5.62	
R postcentral gyrus		52	−22	48	3.45	5.56	
R insula lobe		42	14	−14	3.35	5.33	
R supramarginal gyrus		32	−38	44	3.32	5.27	
R postcentral gyrus		38	−28	40	3.09	4.79	
R precentral gyrus		46	−14	56	2.77	4.18	
L superior temporal gyrus	10 449	−40	−32	12	>8	71.04	<0.001
L insula lobe		−32	26	6	6.62	15.93	
L superior temporal gyrus		−52	2	−2	5.62	11.86	
L inferior frontal gyrus		−34	6	26	4.59	8.49	
L inferior frontal gyrus		−44	16	22	4.30	7.66	
L inferior frontal gyrus		−48	10	16	4.01	6.89	
L inferior frontal gyrus		−56	28	18	3.97	6.79	
L inferior frontal gyrus		−40	8	16	3.91	6.64	
L precentral gyrus		−48	−4	48	3.86	6.50	
L inferior frontal gyrus		−36	38	12	3.85	6.50	
L precentral gyrus		−46	4	32	3.62	5.93	
L inferior frontal gyrus		−48	34	6	3.48	5.63	
L precentral gyrus		−48	0	40	3.29	5.21	
L inferior frontal gyrus		−48	34	16	3.25	5.13	
L middle frontal gyrus		−36	34	28	3.25	5.11	
L cuneus	6227	−16	−56	22	4.79	9.08	<0.001
L precuneus		−14	−58	30	4.70	8.81	
L middle occipital gyrus		−36	−74	30	4.70	8.81	
L inferior parietal lobule		−30	−48	42	4.60	8.50	
L superior parietal lobule		−22	−64	44	4.53	8.31	
L middle occipital gyrus		−22	−62	34	4.29	7.64	
R middle occipital gyrus		40	−70	30	4.29	7.64	
R precuneus		6	−56	20	4.28	7.60	
R angular gyrus		50	−60	26	4.16	7.28	
L inferior parietal lobule		−36	−40	40	4.11	7.16	
R superior parietal lobule		16	−60	50	4.07	7.03	
L inferior parietal lobule		−44	−40	42	4.06	7.02	
L precuneus		−4	−60	20	4.00	6.88	
R superior parietal lobule		26	−56	46	3.65	6.02	
L cuneus		−8	−72	30	3.34	5.31	
Cerebellar vermis	579	2	−38	−6	4.45	8.09	0.01
R thalamus		22	−18	−8	3.78	6.31	
R thalamus		12	−26	−6	3.46	5.58	
R thalamus		10	−10	2	3.34	5.32	
R hippocampus		30	−18	−16	3.34	5.31	
L posterior cingulate cortex		−8	−42	12	3.15	4.92	

Note: The results listed in the table (*F* contrast, one-way repeated-measures ANOVA) are presented at an uncorrected threshold of *P* < 0.005 peak level, corrected with nonstationary correction of *P* < 0.05 at cluster level. R, right; L, left. We report a maximum of 15 gray matter local maxima (that are more than 8 mm apart) per cluster.

**Figure 3.**
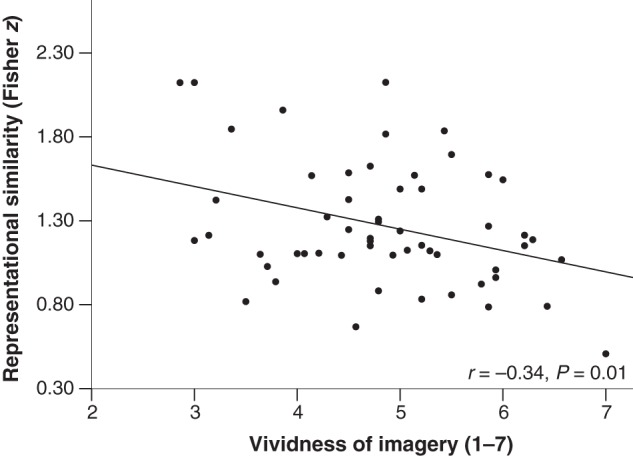
Association between lower representational similarity of functional responses to different types of heard sounds in SMA (i.e., higher specificity/fidelity) and higher reported vividness of auditory imagery.

To assess whether regions involved in auditory imagery co-localized with those involved in the processing of heard auditory information, analyses were conducted looking at hemodynamic responses within the clusters in which gray matter volume correlated with vividness imagery ratings in the main VBM study. Using small volume correction, we found that the left SMA (cluster presented in Fig. 1) shows significant modulation of the neural response as a function of sound type (MNI coordinate for peak voxel: *x* = −8, *y* = −2, *z* = 62, *F*_(4,220)_ = 5.51, *Z* = 3.43, *P*_FWE_ = 0.03), suggesting that this region plays a role in imagery and in the processing of heard information. Crucially, we additionally conducted a representational similarity analysis (see Materials and Methods) to examine whether this co-localization in SMA reflects the operation of converging mechanisms. Activity patterns associated with each pair of intelligible vocal sound types were compared (linear correlations, *n* = 10), the pairs were assembled, and an average similarity was computed for each participant (*M =* 0.83; SD *=* 0.1; range = 0.47–0.97); this analysis was conducted within a sphere with 12 mm radius (925 voxels). In keeping with the hypothesis that mechanisms are shared, lower neural similarity between vocal sounds correlated with higher vividness of auditory imagery, that is, participants with higher specificity of neural representations during the processing of heard auditory information also reported experiencing more vivid mental auditory images (*r* = −0.34, *P* = 0.01; after regressing out demographic and cognitive variables, as in the main VBM study, *r* = −0.42, *P* = 0.001). This association is shown in Figure 3. A further model was conducted to examine whether the magnitude of the distinction between intelligible vocal sounds and the condition of unintelligible sounds was also associated with imagery. We computed an average of similarity of neural responses between each type of vocal sound and rotated sounds for each participant (linear correlations, *n* = 5; neutral sounds vs. rotations, laughter vs. rotations, etc.), and found a significant correlation between lower similarity and higher vividness of auditory imagery (*r* = −0.42, *P* = 0.001; after regressing out demographic and cognitive variables, *r* = −0.50, *P* < 0.001). This finding suggests that participants reporting higher vividness of mental auditory images not only show higher representational specificity of different intelligible vocal sounds, as they also appear to show sharper distinctions between vocal and unintelligible sounds within SMA.

Perceptual–functional modulations as a function of sound type were also found in three of the clusters selected from the imagery meta-analysis (and in which the amount of gray matter predicted vividness ratings in the current study; see Table 1): one in left SMA as well (MNI coordinate for peak voxel: *x* = −8, *y* = 0, *z* = 60, *F*_(4,220)_ = 5.52, *Z* = 3.43, *P*_FWE_ = 0.03), one in the left inferior parietal lobule (MNI coordinate for peak voxel: *x* = −32, *y* = −48, *z* = 44, *F*_(4,220)_ = 8, *Z* = 4.42, *P*_FWE_ < 0.001), and one in the right superior parietal lobule (MNI coordinate for peak voxel: *x* = 16, *y* = −50, *z* = 50, *F*_(4,220)_ = 7.03, *Z* = 4.07, *P*_FWE_ < 0.004). Representational similarity analyses were also conducted for these clusters. Correlations between representational similarity and vividness of imagery approached significance for the left SMA cluster (*r* = −0.23, *P* = 0.09; after regressing out demographic and cognitive variables, *r* = −0.33, *P* = 0.01), but they were nonsignificant for the left inferior parietal (*r* = −0.10, *P* = 0.48; after regressing out demographic and cognitive variables, *r* = −0.10, *P* = 0.45) and right superior parietal clusters (*r* = −0.12, *P* = 0.39; after regressing out demographic and cognitive variables, *r* = −0.09, *P* = 0.5).

These results suggest that brain regions whose structure predicts individual differences in auditory imagery, notably the SMA and parietal systems, are also engaged by processing of auditory information. A direct association between imagery and sensory-based processing could however be established for the SMA only.

### Links Between Auditory and Visual Imagery

**Figure 4.**
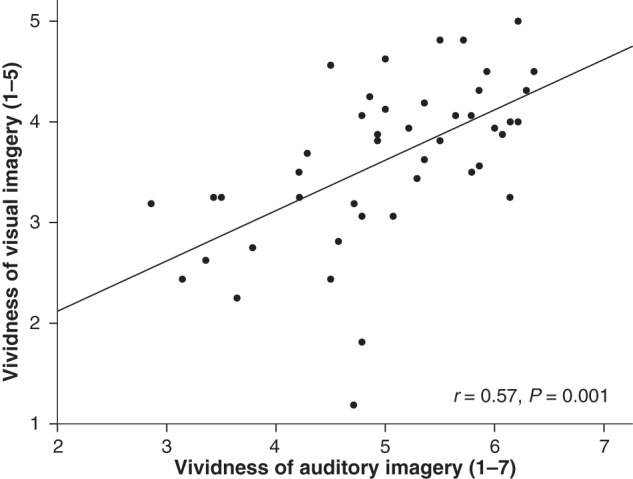
Association between vividness of visual and auditory imagery. Higher vividness corresponds to higher ratings for auditory and visual imagery.

From the results described so far, it cannot be determined whether the underlying mechanisms are specialized for auditory information or whether they are supramodal in nature to some extent. To shed light on this question, we investigated behavioral and neural correlations between auditory and visual imagery. Considerable individual differences were obtained in visual imagery ratings (VVIQ): ratings ranged between 1.19 and 5 (5 = maximally vivid; *M =* 3.63; SD = 0.81). A strong correlation was found between reported vividness of auditory and visual imagery (*r* = 0.57, *P* < 0.001; see Fig. 4), a correlation that remains significant after regressing out demographic and cognitive variables (*r* = 0.53, *P* < 0.001). This indicates that participants who report generating highly vivid auditory images also report generating highly vivid visual images. Additionally, higher vividness of visual imagery correlated with gray matter volume within the SMA cluster previously shown to correlate with vividness of auditory imagery (in the whole-brain VBM analysis, Fig. 1; MNI coordinate for peak voxel: *x* = 4, *y* = −12, *z* = 72, *t*_(1,39)_ = 3.25, *Z* = 3.04, *P*_FWE_ = 0.048). To investigate whether this association reflects unique variance associated with visual imagery (i.e., independent of auditory imagery), we correlated gray matter residuals with visual imagery while regressing out variability in vividness of auditory imagery; the partial correlation coefficient was not significant (*r* = 0.03, *P* = 0.82). No other associations between gray matter and visual imagery were found, both in whole-brain analysis and after small volume corrections within other regions implicated in imagery (Table 1).

## Discussion

The present study examined the structural basis of interindividual differences in auditory imagery, and how these differences reflect commonalities in sensory-based processing and mechanisms that are involved in imagery across modalities. We present 4 novel findings. First, using VBM, we established that differences among individuals in the reported vividness of auditory imagery are predicted by the amount of gray matter in the SMA, inferior and superior parietal lobules, medial superior frontal gyrus, and middle frontal gyrus. Second, in an fMRI experiment, these SMA, inferior, and superior parietal sites were also modulated as a function of vocalization type during the processing of heard auditory information. Third, a representational similarity analysis revealed that higher representational specificity of different types of vocal sounds within SMA predicts higher vividness of mental auditory images, a result that directly links sensory- and imagery-based processing. Fourth, cross-modal interactions were found at behavioral and structural levels: self-report behavioral measures of auditory and visual imagery were correlated, and individual differences in visual imagery were also predicted by the amount of gray matter in SMA. These findings are discussed in the next paragraphs.

Although a number of studies have shown that temporal, parietal, motor, and prefrontal regions are typically active during auditory imagery tasks (e.g., Shergill et al. 2001; Herholz et al. 2012; Zvyagintsev et al. 2013), relatively little was known about which of these systems (and how) predict variability in behavioral correlates of imagery (Daselaar et al. 2010; Zatorre et al. 2010; Herholz et al. 2012). Consistent with previous performance-based (Janata 2012) and self-report evidence (Pfordresher and Halpern 2013; Gelding et al. 2015), our behavioral measure revealed that auditory imagery varies considerably across individuals. Crucially, here we show for the first time that this variability relates to differences in the local structure of gray matter. The association between higher perceived vividness of auditory images and increased gray matter volume in SMA adds to functional research reporting activity in this region during auditory imagery tasks requiring the imagination of tunes (Herholz et al. 2012; Zvyagintsev et al. 2013), timbre of musical instruments (Halpern et al. 2004), verbal information (Shergill et al. 2001; Linden et al. 2011), and anticipating sound sequences (Leaver et al. 2009). It is also in accord with exploratory results showing a correlation between the magnitude of BOLD responses in SMA and higher vividness ratings during a task involving imagery of familiar melodies (Zvyagintsev et al. 2013). The other regions in which the amount of gray matter predicted vividness of imagery, namely left inferior and right superior parietal cortices, right medial superior frontal gyrus, and left middle frontal gyrus were recently highlighted by a meta-analysis as part of a core imagery network (McNorgan 2012), and they have been shown to be engaged across different kinds of auditory imagery tasks (Shergill et al. 2001; Zatorre et al. 2010; Linden et al. 2011; Zvyagintsev et al. 2013).

Extending previous findings, the present study demonstrates not only that these systems are functionally implicated in imagery, but also that their structural features are diagnostic of behavioral outcomes. Our results were obtained using an off-line self-report measure that covers ecologically valid and diverse scenarios, which was completed in comfortable conditions, that is, not constrained by being inside an MRI scanner. Importantly, this measure has been shown to index mechanisms that are also involved in active, performance-based, imagery tasks. It correlates with brain activity during active imagery tasks (reversal of melodies, Zatorre et al. 2010; imagery of familiar tunes, Herholz et al. 2012), and with performance levels in behavioral tasks: pitch discrimination (Pfordresher and Halpern 2013), and detection of mismatches between a probe note and the last note of an imagined sequence (Gelding et al. 2015). This adds to the mounting evidence that self-report measures provide rich information about individual differences in an array of cognitive processes, and can significantly relate to brain structure (Kanai et al. 2011; Banissy et al. 2012). For instance, Kanai et al. (2011) observed that a self-report measure of everyday distractibility correlates with gray matter volume in the left superior parietal cortex, as well as with a performance-based measure of attention capture. Because of the characteristics of these measures, however, one concern regards the potential confounding effects of participants' abilities to report on their own experience (metacognition), or of their general cognitive ability (e.g., working memory; attention). Our results are unlikely to be reducible to such processes: we controlled for performance on a short-term memory task that correlates with working memory and intelligence (Colom et al. 2008), and we showed that associations between vividness and brain structure remain significant after accounting for responses on the other BAIS subscale focusing on control of imagery, which would load on the same nonspecific metacognitive factors. Moreover, the ability to introspect about self-performance correlates with gray matter volume in the right anterior prefrontal cortex (Fleming et al. 2010), a region involved in high-level control of cognition and in the integration of perceptual information with decision output. This region does not overlap with those identified here.

It was unexpected that we did not find an association between auditory imagery and the structure of STG, even after small volume correction. Auditory association areas were previously found to be more strongly activated during auditory versus others forms of imagery (Zvyagintsev et al. 2013), and they have been assumed to support the reconstruction of auditory-like representations (Janata 2001; Kraemer et al. 2005; Lange 2009; Navarro-Cebrian and Janata 2010a). It was further reported that the magnitude of BOLD responses within these areas predicts vividness ratings during imagery (Daselaar et al. 2010; Herholz et al. 2012; Zvyagintsev et al. 2013), even though this finding is not always replicated (Leaver et al. 2009). Our null result does not weaken the well-established idea that STG plays a functional role for auditory imagery, but it suggests that macroscopic gray matter differences in this region are not a source of interindividual variability in the behavioral measure used here. This may indicate that anterior control and sensorimotor systems have a more prominent role than posterior auditory ones for individual differences in imagery, or that the structural predictors partly depend on the specific task demands. Indeed, there is fMRI and electrophysiological evidence that activity in auditory association areas is preceded and controlled by more anterior regions during imagery. Herholz et al. (2012) found increased connectivity between STG and prefrontal areas for imagery versus perception of tunes. Linden et al. (2011) showed that activity in SMA precedes that of auditory areas during voluntary imagery, and that this timing is impaired during hallucinations (lack of voluntary control). In the visual domain, Borst et al. (2012) showed that activity in frontal regions precedes that of more posterior regions, namely of occipital cortex, in a scene imagery task. In addition to being activated first, responses in frontal regions also predicted reaction times on the scene imagery task (consisting of judging whether a visually presented fragment of the scene was mirrored or not), while other regions did not. Concerning possible task effects, the self-report measure used here focuses on perceived vividness and on the sense of control over auditory images; it remains to be seen whether individual differences in performance-based imagery tasks requiring a fine-grained analysis of sound representations would reveal a structural role of STG (e.g., judging whether a probe note is mistuned or not, Janata and Paroo 2006; Navarro-Cebrian and Janata 2010a, 2010b).

The amount of gray matter in SMA was the most robust predictor of vividness of auditory imagery, an effect found both in whole-brain analysis and in the ROI analyses based on the meta-analysis of functional studies on imagery (McNorgan 2012). Supporting the hypothesis that imagery partly engages the network that responds to heard auditory information, we also observed that this region was modulated by vocal sound category in the fMRI study, along with other regions that are typically engaged by intelligible vocal information, such as bilateral STG (e.g., Warren et al. 2006; Okada et al. 2010; Evans et al. 2014; McGettigan et al. 2015). Our functional results are consistent with previous work reporting the engagement of motor systems during the processing of vocal information (Warren et al. 2006; McGettigan et al. 2015). We focus on vocalizations only, but these systems seem to be recruited by complex sounds more generally (Scott, McGettigan, et al. 2009), such as music (Zatorre et al. 2007; Herholz et al. 2012), degraded speech (Mattys et al. 2012), and sounds derived from human actions like kissing or opening a zipper (Gazzola et al. 2006). Regarding the links between imagined and heard information, although previous studies observed common activations in SMA using linguistic and musical stimuli (Zatorre et al. 1996; Herholz et al. 2012), here we went a step further: we show co-localization across structural and functional levels and, crucially, we provide the first evidence for co-variation between vividness of auditory imagery and specificity of neural representations of heard auditory information within this region. Such an association is central to the argument that co-localization reflects the operation of similar mechanisms.

The SMA provides a crucial link between perception and action, and its functional attributes facilitate many cognitive and motor processes. It is involved in aspects of action including planning, initiation and inhibition, in learning new associations between stimuli and motor responses, in cognitive control processes such as switching between motor plans, and in the passive observation of grasping actions and emotional expressions (Warren et al. 2006; Kleber et al. 2007; Nachev et al. 2008; Mukamel et al. 2010). Mukamel et al. (2010) recorded single-neuron responses in humans during the observation and execution of grasping actions and facial gestures, and found that a significant number of neurons in SMA responded to both conditions, revealing sensorimotor properties. As for the structure of SMA, previous studies demonstrated that it may vary across individuals as a function of motor learning and expertise: there is longitudinal evidence of increments in the volume of gray matter during 6 weeks of learning of a complex motor task (Taubert et al. 2010), as well as cross-sectional evidence of expertise-related structural differences in gymnasts (Huang et al. 2013) and ballet dancers (Hänggi et al. 2010). That sensorimotor systems respond to different types of complex auditory information, even when participants are not required to perform or plan any movements, may reflect the automatic engagement of some level of sensorimotor simulation. Processing and evaluating complex sounds—human vocalizations, in the case of the current study—would involve the activation of motor representations that link sensory information to actions related to the production of those sounds (Gazzola et al. 2006; Warren et al. 2006; Scott, McGettigan, et al. 2009; Scott et al. 2014; McGettigan et al. 2015). We argue that the same mechanism of covert simulation may support auditory imagery—an imagery-to-action pathway. Accessing auditory–motor representations may be central for the generation of different types of mental auditory images, such as vocal and musical ones (Halpern and Zatorre 1999; Meteyard et al. 2012; Zvyagintsev et al. 2013), and the structure of sensorimotor systems may be a determinant of the efficiency of this mechanism. The perceived vividness of mental images and the representational specificity of heard information would both be shaped by how efficiently relevant sensorimotor information is retrieved.

Such an imagery-to-action pathway is unlikely to be specialized to auditory information, as other forms of imagery (e.g., visual, motor) may also have associated action components and engage sensorimotor processes to some extent. Indeed, activity in SMA is observed in functional studies conducted on nonauditory modalities of imagery (Guillot et al. 2009; Borst et al. 2012; McNorgan 2012; Hétu et al. 2013; Zvyagintsev et al. 2013). Furthermore, SMA is similarly active during motor imagery and execution, suggesting that movement sensations are simulated during motor imagery (Naito et al. 2002; Ehrsson et al. 2003; Hanakawa et al. 2003). The same was suggested in the visual domain (Grèzes and Decety 2002; Solodkin et al. 2004; Zacks 2008; Mukamel et al. 2010). However, despite the suggestive evidence of cross-modal commonalities in the mechanisms supporting imagery, only rarely have different modalities been directly compared (Halpern et al. 2004; Solodkin et al. 2004; Daselaar et al. 2010). We established that participants reporting highly vivid auditory images also report experiencing highly vivid visual images. That vividness of visual imagery is reflected in differences in gray matter volume in SMA, paralleling the findings for auditory imagery, suggests that converging sensorimotor simulation processes may operate across modalities. These commonalities may further reflect the fact that everyday imagery often involves multisensory components, that is, mental images are frequently not confined to one single modality (Hubbard 2013). Even in an experimental setting in which the task requires participants to focus on a particular modality, components from other modalities may be spontaneously retrieved. When asked to generate an image of an auditory scene, for instance, concurrent visual and kinesthetic images might spontaneously appear (e.g., when imagining the cheer of the crowd as a player hits the ball—one of the BAIS items—individuals may also generate a visual image of the crowd in a stadium). In future studies, it would be interesting to examine whether the diversity of components retrieved for an auditory or visual scene may actually contribute to enhance the impression of vividness.

To conclude, the present study forms the first demonstration that interindividual differences in auditory imagery have a signature in brain structure, adding to the growing body of evidence that individual differences can be an invaluable source of information to link behavior and cognition to brain anatomy. Building upon prior functional neuroimaging studies, our results establish a role for the structure of parietal, prefrontal, and sensorimotor systems (in particular SMA) in supporting auditory imagery. In SMA, we further established links between auditory imagery, processing of heard vocal information, and visual imagery. We argue for sensorimotor simulation as a candidate mechanism for such commonalities. Future investigations could extend this work to refine the exploration of converging computations between imagery and auditory processing, for example, by including different types of perceived and imagined sounds that afford a wider range of variability in terms of the accessibility of relevant sensorimotor representations. Our focus was on links between heard human vocal information and auditory imagery mostly for voices and music (the main domains covered by the BAIS). Further work will also need to specify the microstructural basis of the large-scale anatomical differences reported here, and to determine how they are shaped by environmental and genetic factors.

## Funding

This work was supported by a Wellcome Trust Senior Research Fellowship (WT090961MA) awarded to Sophie Scott. During the planning and execution of this project, César Lima was funded by a postdoctoral fellowship from the Portuguese Foundation for Science and Technology (SFRH/BPD/77189/2011), and Andrea Halpern was partly funded by a Leverhulme Visiting Professorship. Funding to pay the Open Access publication charges for this article was provided by The Wellcome Trust.
